# Measuring multimorbidity in older adults: comparing different data sources

**DOI:** 10.1186/s12877-019-1173-4

**Published:** 2019-06-14

**Authors:** Samantha Gontijo Guerra, Djamal Berbiche, Helen-Maria Vasiliadis

**Affiliations:** 1 Centre de recherche Charles-Le Moyne - Saguenay–Lac-Saint-Jean sur les innovations en santé (CR-CSIS), Longueuil, QC Canada; 2 Université de Sherbrooke, Campus Longueuil, 150 Place Charles-Le Moyne, Longueuil, QC J4K 0A8 Canada; 3 Department of Community Health Sciences, Faculty of Medicine and Health Sciences, Université de Sherbrooke, Longueuil, QC Canada

**Keywords:** Chronic conditions, Multimorbidity, Prevalence, Epidemiology, Self-report, Health administrative data, Primary health care, Older adults, Data sources, Agreement between sources

## Abstract

**Background:**

Multimorbidity is a global health issue, particularly for older adults in the primary care setting. An adequate portrayal of its epidemiology is essential to properly identify and understand the health care needs of this population. This study aimed to compare the differences in the prevalence of selected chronic conditions and multimorbidity, including its associated characteristics, using health survey/self-reported (SR) information only, administrative (Adm) data only and the combined (either) sources.

**Methods:**

This was a secondary analysis of survey data from the first cycle of the *Longitudinal Survey on Senior’s Health and Health Services Use* linked to health-Adm data. The analytical sample consisted of 1625 community-dwelling older adults (≥65 years) recruited in the waiting rooms of primary health clinics in a selected administrative region of the province of Quebec. Seventeen chronic conditions were assessed according to two different data sources. We examined the differences in the observed prevalence of chronic conditions and multimorbidity and the agreement between data sources.

**Results:**

The prevalence of each of the 17 chronic conditions ranged from 1.2 to 68.7% depending on the data source. The agreement between different data sources was highly variable, with kappa coefficients (κ) ranging from 0.05 to 0.73. Multimorbidity was very high in this population, with an estimated prevalence of up to 95.9%. In addition, we found that the association between sociodemographic and behavioural factors and the presence of multimorbidity varied according to the different data sources and thresholds.

**Conclusions:**

This is the first study to simultaneously investigate chronic conditions and multimorbidity prevalence among primary care older adults using combined SR and health-Adm data. Our results call attention to (1) the possibility of underestimating cases when using a single data source and (2) the potential benefits of integrating information from different data sources to increase case identification. This is an important aspect of characterizing the health care needs of this fast-growing population.

**Electronic supplementary material:**

The online version of this article (10.1186/s12877-019-1173-4) contains supplementary material, which is available to authorized users.

## Background

Multimorbidity (MM), the co-occurrence of multiple chronic conditions in an individual [1], is a prominent public health issue that is attracting increasing interest among researchers, practitioners and decision-makers from a variety of disciplines and fields. The primary care setting undertakes a substantial part of the management of multimorbid patients [2–4], and although MM is not observed exclusively in older adults, this group is particularly affected [5, 6]. Since MM is undoubtedly a central element of the framework describing the needs of older adults, an adequate description of the epidemiology of concurrent physical and mental health conditions [3, 4, 6–8] is essential in informing the responsiveness of health systems [4, 9].

The designs of epidemiological MM studies vary greatly, as do their results in terms of prevalence (ranging from 3.5 to 100%) [10] and associated outcomes [11–14]. This can be partly explained by the lack of consensus concerning the conceptual or operational definitions (i.e., the length of the list of conditions, cut points, etc.) [10, 15, 16], choice of study populations [17, 18] and data sources [19–21]. It is not always clear, however, which data source is most appropriate to use in epidemiological studies [22].

The objective of comparing different data sources has been well explored in studies considering individual diagnoses (e.g., diabetes, hypertension, cancer) and in those including a relatively small number of diseases [23–26]. These studies have predominantly compared self-reports to chart reviews, medical examinations or practitioner reports [22, 27, 28]. Recently, survey/self-reported (SR) and administrative (Adm) data have been increasingly used in health research [23]. As suggested by Bayliss and colleagues [29], these data sources represent the subjective (patient’s perspective) and objective (health systems’ perspective) measurements that must be taken into account to make a more comprehensive assessment of morbidity.

We identified only two studies that simultaneously compared the prevalence of chronic conditions and MM using SR and Adm data [30, 31] with one study [30] reporting prevalence estimates measured with the combined (either) data sources. The use of combined data sources in epidemiological studies has been suggested as a promising approach since it allows the perspectives of both the patient and the health system to be taken into account [32–34]. To date, no previous study of this nature (i.e., comparing and combining SR and health-Adm data) has been conducted among older populations of primary health care users, a well-known priority population in terms of MM. Using SR and health-Adm data, individually or combined, the current study aimed to (1) compare the prevalence of selected chronic conditions and MM; (2) examine the agreement between these data sources on selected chronic diseases; (3) examine the factors associated with MM as a function of data sources and (4) draw out implications for policy and future research.

## Methods

### Study design and setting

This was a secondary analysis of data from the first cycle of the *Longitudinal Survey on Senior’s Health and Health Services Use* (Enquête sur la santé des aînés-Services or ESA-S) linked to health-Adm data. The participants consisted of French-speaking, community-dwelling older adults aged 65 years and over recruited between 2011 and 2013 in the waiting rooms of primary health clinics in a selected administrative region of the province of Quebec. The purpose of the ESA-S survey was to document the episodes of psychological distress and the factors associated with the use of health services for mental health problems in older people. More details on the ESA-S survey methodology can be obtained elsewhere [35]. Eligible candidates were contacted for in-home interviews. To minimize information bias associated with mild to severe cognitive impairment, individuals scoring less than 22 on the Mini-Mental State Examination (MMSE) [36, 37] were excluded at the beginning of the interview. Of the 1811 individuals contacted, 46 scored ≤21 on the MMSE and did not continue the interview. Since the health-Adm and SR data linkage was not achieved for 140 of the 1765 participants who completed the interviews, the analytical sample for this study included 1625 participants.

### Data source and collection

Survey data were collected by trained professionals. During a face-to-face interview, participants responded to a self-reported, computer-administered questionnaire (the ESA-S Q) aimed at collecting sociodemographic, behavioural and clinical information. They were first invited to sign an informed consent form. In addition, each participant was asked to authorize the research team to access their health-Adm data from ministerial databases on claim-based medical services and hospitalization, the Régie de l’Assurance Maladie Québec (RAMQ) and the Maintenance et exploitation des données pour l’étude de la clientèle hospitalière (MED-ÉCHO), respectively. These databases include information allowing, among other things, the identification of diagnoses (according to the International Statistical Classification of Diseases and Related Health Problems, 9th and 10th revisions – ICD-9/ICD-10) and the determination of whether medical care was provided during a hospitalization, an outpatient or an emergency department (ED) visit. Health-Adm and SR databases were linked using the personal health insurance number (Numéro d’assurance maladie – NAM), a unique 10-digit identifier for each beneficiary. In the Canadian province of Quebec, residents are covered by a universal health insurance programme administered by the RAMQ.

### Measurements and study variables

#### Chronic conditions

##### Self-reported

The presence of SR chronic conditions was ascertained based on participants’ answers (yes/no) to the following question, *“To your knowledge, and according to a physician, do you have […]”.* Briefly*,* participants were invited to inform if they had been diagnosed with one or more chronic conditions out of a list of 15: dermatologic conditions, arthropathy, cancer, headaches, diabetes, liver disease, hyperlipidemia, hypertension, cardiovascular disease/atherosclerosis, eye diseases, respiratory tract disease (i.e., bronchitis, asthma, emphysema, persistent cough), chronic urinary tract problem, thyroid problems, gastrointestinal diseases, and musculoskeletal conditions (i.e., chronic neck/back pain). Moreover, the presence of depressive and anxiety symptoms was ascertained using the ESA-S Q Mental-Diagnostic Module based on the Diagnostic and Statistical Manual of Mental Disorders, 5th edition (DSM- 5) and other literature sources [38–40]. The presence of depressive (major, minor and subclinical) and anxiety (specific phobia, social phobia, agoraphobia, panic disorder, and generalized anxiety disorder—GAD) conditions was then defined [38–40]. We defined common mental health disorders as the presence of anxiety and/or depressive conditions. Last, participants were also asked to report their weight and height so that their body mass index (BMI) could be calculated and self-reported obesity (defined as a BMI ≥ 30) [41] ascertained.

##### Physician diagnosis based on administrative records

Two health-Adm databases (RAMQ medical services claim-based and MED-ÉCHO hospital stay) were used to identify participants’ chronic conditions. The ICD-9/ICD-10 codes were used to extract information from these databases (over a three-year period prior to the interview) for each of the 17 conditions reported by the participants. The selection of the ICD-9/10 codes was mostly guided by a list proposed by a group of experts from the International Research Community on Multimorbidity (IRCMo). This list was also completed by literature review [40, 42–45] in the case of disease categories that were not included in the expert group’s list (e.g., eye disease, dermatologic disease and chronic headaches). An additional table shows the list of 17 chronic condition categories and the corresponding ICD-9 and ICD-10 codes (see Additional file 1).

##### Combined data sources

Each of the 17 chronic conditions was also assessed using combined data sources (either SR or Adm).

#### Multimorbidity (MM) variables

For each of the three datasets (i.e., SR only, Adm only, combined sources/either SR or Adm), a count of conditions was created by summing up the total number of chronic conditions, ranging from 0 to 17. Six MM variables were then operationalized according to (1) two different cut-offs frequently used in the literature, i.e., more than 2 (MM2) and more than 3 (MM3) conditions [11, 12, 46] and (2) the type of data source. They were named as follows: MM2_SR, MM2_Adm, MM2_Either, MM3_SR, MM3_Adm, and MM3_Either.

#### Other study variables

We also assessed participants’ gender (male; female), age groups (65–69, 70–74; 75–79; 80–84; 85–89; ≥90), education in years (≤7; 8–12; ≥13), annual household income in $CAN (< 25,000; ≥ 25,000), current marital status (with partner; without partner), perceived mental and physical health (excellent/very good; good; fair/poor), and smoking habits (never; former; current smoker). Social support (SS) was assessed using three dichotomous (0/1) questions about the availability of someone (1) to whom one can confide on various issues, (2) who could provide instrumental help and (3) who could provide emotional support. A summative score, ranging from zero to 3, was then calculated (higher scores indicating higher SS). The number of outpatient medical consultations and ED visits for any reason was obtained from the RAMQ medical registry, and the number of hospitalizations was obtained from the MED-ÉCHO registry, for a period of 3 years prior to the interview. For this study, outpatient services delivered on the same day for the same person by two or more different medical practitioners were computed as different visits. An ED visit was identified as all claims for medical acts performed in this department on the same date, and hospitalization was defined as each hospital inpatient stay lasting more than 24 h.

### Statistical analysis

We used general descriptive statistics, and the results were expressed as absolute frequency and proportions or mean ± standard deviation (SD). Bivariate analyses were performed using the chi-square test for categorical variables and Student’s t-test for continuous variables. All statistical analyses were conducted using SPSS 25 with a statistical significance alpha level of 0.05.

Differences in the observed prevalence of chronic conditions and of MM among data sources were assessed using Cochran’s Q Test [47]. We calculated the relative percent (%) difference of proportions obtained with single data sources (SR vs Adm) using the following formula: [(A-B)/A] *100%, where (A) corresponds the higher proportion and (B) corresponds to the lower proportion obtained with an individual dataset (whether Adm or SR). The relative percent (%) increase in prevalence that occurred when data sources were combined [48] was calculated using the following formula [(C-A) /A] *100%, where (C) corresponds to the proportion obtained with combined data sources and (A) corresponds the higher proportion obtained with an individual dataset (whether Adm or SR).

Agreement between the SR and Adm data sources on chronic conditions was assessed using kappa statistics (κ), positive (PA) and negative (NA) agreement, prevalence index (PI), bias index (BI) and prevalence-adjusted and bias-adjusted κ (PABAK). The PI measures the difference between the probability of “yes” and “no” categories for two observations or observers, while the BI describes how much the two observations or observers differ on the proportion of positive results. For any 2 × 2 table, these indexes can be estimated by PI = (a-d)/N and BI = (b-c)/N [49]. The range of possible values of κ is from − 1 to 1, and their suggested interpretations are as follows: ≤ 0 (no agreement); 0.01 to 0.20 (none to poor), 0.21 to 0.40 (fair), 0.41 to 0.60 (moderate), 0.61 to 0.80 (substantial), and 0.81 to 1.00 (almost perfect agreement) [50, 51].

All data were anonymized. Missing data were estimated using an appropriate method for the imputation of categorical data. This method maximizes the consistency of the completed data as measured by Guttman’s squared correlation ratio, as suggested by van Buuren and van Rijckevorsel [52].

### Ethics approval

The research ethics board of the CIUSSS de l’Estrie—CHUS reviewed and approved the ESA-S project [#2012–03]. Access to the health-administrative database was approved by the Commission d’accès à l’information du Québec (CAIQ).

## Results

Comparisons between individuals with (*n* = 1625) and without (*n* = 140) available Adm data did not show statistically significant differences with respect to gender (*p* = 0.08), age group (*p* = 0.13), income (*p* = 0.06), marital status (*p* = 0.65), smoking habit (*p* = 0.71), perceived mental (*p* = 0.99) or physical (*p* = 0.40) health and MMSE score (*p* = 0.17). However, those for whom matched health-Adm data were not possible had a significantly lower level of education (*p* = 0.04), fewer self-reported chronic conditions (*p* < 0.01) and less social support (*p* < 0.01). The overall sample characteristics are shown in Table 1.

**Table 1:** Sociodemographic and behavioural characteristics of the study population

Characteristics	Total sample *n* = 1625	
	n	%
Gender		
M	704	43.3
F	921	56.7
Age groups		
65–69	532	32.7
70–74	494	30.4
75–79	334	20.6
80–84	185	11.4
85–89	70	4.3
90 et +	10	0.6
Education (in years)		
< 7	409	25.2
8–12	714	43.9
≥13	502	30.9
Income		
0–25,000	555	34.2
≥ 25,000	1070	65.8
Marital status		
With partner	1030	63.4
Without	595	36.6
Perceived mental health		
Excellent/very good	1130	69.5
Good	391	24.1
Fair/poor	104	6.4
Perceived physical health		
Excellent/very good	896	55.1
Good	469	28.9
Fair/poor	260	16
Smoking		
Never	616	37.9
Former	869	53.5
Current	140	8.6
Social support (mean ± SD)	2.82 (0.51)	
Number of outpatient visits (mean ± SD)	25.66 (22.66)	
Number of ED visits (mean ± SD)	1.27 (2.09)	
Number of hospitalizations (mean ± SD)	0.45 (1.00)	

*Abbreviation*: *SD* Standard deviation

**Fig. 1:**
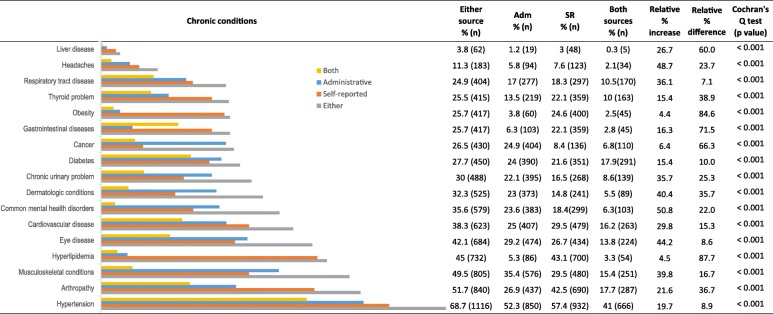
Prevalence of chronic conditions by data sources and estimated relative percentage increase

The prevalence of each of the 17 chronic conditions ranged from 3 to 57.4%, 1.2 to 52.3% and 3.8 to 68.7% when using SR data only, Adm data only and either data sources, respectively (Fig. 1). Cochran’s Q test indicated statistically significant differences among the three proportions (SR vs Adm vs either data sources) for all conditions. Our analysis showed relative percent differences ranging from 7.1 to 87.7%, and when combining data sources, estimations of proportions increased from 4.4 to 50.8% for all conditions.

As Table 2 shows, concordance was highly variable, and the κ coefficients ranged from 0.05 to 0.73 (poor to substantial agreement), whereas the PABAK varied from 0.16 to 0.93. With the exception of hypertension, all chronic conditions had higher negative agreement than positive agreement.

**Table 2:** Summary of agreement analysis for chronic conditions

Chronic condition	Kappa (95% CI)	Interpretation	Positive Agreement (PA)	Negative Agreement (NA)	Prevalence Index (PI)	Bias Index (BI)	PABAK
Liver disease	0.13 (0.02 to 0.25)	Poor	0.15	0.98	0.96	0.02	0.93
Headaches	0.27 (0.18 to 0.35)	Fair	0.31	0.95	0.87	0.02	0.82
Respiratory tract disease	0.50 (0.45 to 0.56)	Moderate	0.59	0.91	0.65	0.01	0.71
Thyroid problem	0.48 (0.42 to 0.53)	Moderate	0.57	0.91	0.64	0.09	0.69
Obesity	0.14 (0.10 to 0.18)	Poor	0.20	0.86	0.71	0.21	0.53
Gastrointestinal diseases	0.11 (0.06 to 0.15)	Poor	0.19	0.87	0.71	0.16	0.54
Cancer	0.32 (0.27 to 0.38)	Fair	0.41	0.88	0.67	0.16	0.61
Diabetes	0.73 (0.69 to 0.77)	Substantial	0.79	0.94	0.54	0.02	0.81
Chronic urinary problem	0.32 (0.26 to 0.37)	Fair	0.45	0.87	0.61	0.05	0.57
Dermatologic conditions	0.14 (0.08 to 0.19)	Poor	0.29	0.84	0.62	0.08	0.47
Common mental health disorders	0.12 (0.07 to 0.17)	Poor	0.30	0.81	0.58	0.05	0.41
Cardiovascular disease	0.44 (0.39 to 0.49)	Moderate	0.59	0.85	0.45	0.04	0.56
Eye disease	0.30 (0.25 to 0.35)	Fair	0.50	0.80	0.44	0.02	0.43
Hyperlipidemia	0.05 (0.02 to 0.07)	Poor	0.14	0.72	0.51	0.38	0.16
Musculoskeletal conditions	0.23 (0.18 to 0.28)	Fair	0.48	0.75	0.35	0.06	0.32
Arthropathy	0.27 (0.23 to 0.32)	Fair	0.51	0.74	0.3	0.16	0.32
Hypertension	0.45 (0.40 to 0.49)	Moderate	0.75	0.69	0.1	0.06	0.45

*Abbreviation*: *CI* Confidence interval

The calculated prevalence of MM ranged from 61.9 to 95.9%, and regardless of the operational definition used (MM2 or MM3), combined sources presented a higher prevalence, followed by SR and Adm data. Cochran’s Q test indicated statistically significant differences among the three proportions (*p* < .001). The presence of MM (any definition) was associated with participant age and perceived health. In addition, health service utilization was consistently higher among participants with MM compared to those with no MM. The prevalence of self-reported MM (both cut-offs) was higher among females and participants without partners. These characteristics were also associated with the MM3_either definition, which is MM defined as ≥3 chronic conditions from either data source. Higher income and education levels were associated with a lower prevalence of self-reported MM2 and MM3 (any definition). Smoking was associated with MM only when it was defined under the threshold of 3 conditions (SR only and Adm data only). Social support was significantly lower among certain multimorbid participants (i.e., MM3_SR and MM3_either) (Table 3).

**Table 3:** Prevalence of MM according to different definitions and the participants’ characteristics

Study sample (*n* = 1625)	Multimorbidity					
	Cut-off 2+			Cut-off 3+		
	MM2_SR	MM2_Adm	MM2_either	MM3_SR	MM3_Adm	MM3_either
	86.7% (*n* = 1409)	81.8% (*n* = 1330)	95.9% (*n* = 1558)	72.9% (*n* = 1184)	61.9% (*n* = 1006)	89.2% (*n* = 1449)
Relative % difference	5.6%			15.1%		
Relative % increase	10.6%			22.4%		
Cochran’s Q test	220.95 *p* < .001			483.24 *p* < .001		
Gender						
M	83.2%	81.8%	95.0%	69.2%	61.4%	87.2%
F	89.4%	81.9%	96.5%	75.7%	62.3%	90.7%
	*p* < 0.001	*p* = 0.980	*p* = 0.133	*p* = 0.003	*p* = 0.693	*p* = 0.027
Age groups						
65–69	80.6%	77.8%	93.0%	66.9%	54.1%	83.1%
70–74	89.3%	81.4%	95.7%	74.3%	62.3%	91.1%
75–79	89.5%	85.6%	98.5%	77.2%	67.1%	92.2%
80–84	89.2%	86.5%	98.4%	75.7%	69.7%	95.1%
85–89	92.9%	85.7%	98.6%	77.1%	71.4%	91.4%
90 and +	100.0%	80.0%	100.0%	90.0%	70.0%	90.0%
	*p* = 0.001	*p* = 0.033	*p* = 0.003	*p* = 0.006	*p* < 0.001	*p* < 0.001
Education (in years)						
< 7	90,0%	84.6%	97.3%	79,0%	69.4%	91.9%
8–12	87.1%	81.4%	95.8%	72.7%	59.4%	89.9%
≥ 13	83.5%	80.3%	94.8%	68.1%	59.4%	85.9%
	*p* = 0.014	*p =* 0.221	*p =* 0.169	*p =* 0.001	*p =* 0.001	*p* = 0.009
Income						
0–25,000	90.8%	83.2%	96.9%	80.4%	65.4%	93.2%
≥ 25,000	84.6%	81.1%	95.3%	69.0%	60.1%	87.1%
	*p* < 0.001	*p =* 0.293	*p =* 0.122	*p* < 0.001	*p =* 0.037	*p* < 0.001
Marital status						
With partner	84.6%	82.2%	95.2%	70.8%	61.4%	87.7%
Without	90.4%	81.2%	97.0%	76.5%	62.9%	91.8%
	*p* = 0.001	*p =* 0.595	*p =* 0.091	*p =* 0.013	*p =* 0.549	*p =* 0.011
Perceived mental health						
Excellent/very good	83.5%	79.5%	95,0%	68.2%	58.8%	86.9%
Good	93.1%	87,0%	97.7%	81.8%	68.5%	93.9%
Fair/poor	97.1%	88.5%	99,0%	89.4%	71.2%	96.2%
	*p* < 0.001	*p* = 0.001	*p* = 0.012	*p* < 0.001	*p* < 0.001	*p* < 0.001
Perceived physical health						
Excellent/very good	79.2%	75.8%	93.5%	60.8%	52.3%	83.8%
Good	94.8%	87.2%	98.5%	83.8%	68.7%	94.2%
Fair/poor	97.7%	93.1%	99.2%	94.6%	82.7%	98.5%
	*p* < 0.001	*p* < 0.001	*p* < 0.001	*p* < 0.001	*p* < 0.001	*p* < 0.001
Smoking						
Never	85.9%	79.5%	95.3%	69.2%	58,0%	88.5%
Former	86.5%	83.8%	96.2%	75.0%	65.4%	89.4%
Current	91.4%	80.0%	96.4%	75.7%	57.9%	90.7%
	*p* = 0.212	*p =* 0.096	*p =* 0.646	*p =* 0.031	*p =* 0.009	*p =* 0.702
Social support (mean ± SD)	2.82 (0.51)^a^	2.83 (0.48)^a^	2.82 (0.51)^a^	2.80 (0.53)^a^	2.82 (0.51)^a^	2.82 (0.52)^a^
	*p* = 0.221	*p* = 0.259	*p* = 0.959	*p* = 0.004	*p* = 0.483	*p* = 0.035
Number of outpatient visits (mean ± SD)	27.04 (23.48)^a^	28.58 (23.63)^a^	26.28 (22.83)^a^	28.84 (24.61)^a^	32.21 (25.42)^a^	27.31 (23.24)^a^
	*p* < 0.001	*p* < 0.001	*p* < 0.001	*p* < 0.001	*p* < 0.001	*p* < 0.001
Number of ED visits (mean ± SD)	1.36 (2.18)^a^	1.44 (2.21)^a^	1.30 (2.11)^a^	1.47 (2.28)^a^	1.72 (2.41)^a^	1.37 (2.17)^a^
	*p* < 0.001	*p <* 0.001	*p* < 0.001	*p* < 0.001	*p* < 0.001	*p* < 0.001
Number of hospitalizations (mean ± SD)	0.49 (1.04)^a^	0.52 (1.06)^a^	0.46 (1.01)^a^	0.53 (1.10)^a^	0.65 (1.18)^a^	0.48 (1.03)^a^
	*p* < 0.001	*p* < 0.001	*p =* 0.003	*p* < 0.001	*p <* 0.001	*p* < 0.001

*Abbreviations*: *ED* Emergency department, *MM* Multimorbidity, *SD* Standard deviation

^a^Compared to those without MM (any definition)

## Discussion

Regardless of the definition used, the prevalence of MM was high in this study. This finding is similar to results observed elsewhere [3, 5, 53, 54] that have included older adults in primary care settings and in which the assessment of MM included a list of at least 12 conditions [55]. Our findings add to the growing body of research highlighting MM not only as a major issue in primary care but also as a global health system issue for the care of older populations [43, 56].

To describe the health care needs and to propose appropriate management of care, it is necessary to take into account the heterogeneity of chronic diseases implicated in MM [4, 7]. This is particularly important in older people presenting with coexisting chronic physical and mental health conditions, who are considered high-level complexity groups [7] with poorer health outcomes that require an individualized care approach [56, 57]*.* Our results suggest that integrating information from different data sources increases the identification of the presence of chronic conditions. Interestingly, this study showed that the proportions of common mental health conditions (SR vs Adm) had a relative percentage difference of 22%. Moreover, when both data sources were combined, the estimation increased by 50.8%. These findings align with earlier studies, based on older adults in the US and general population studies in Canada, that propose combining different data sources for improved case identification [58], particularly in neuropsychiatry [48, 59]. Moreover, our study has been one of the first attempts to thoroughly examine the benefits of combining different data sources among a large sample of older adults consulting in primary care settings. This further highlights the important contribution of different data sources in the investigation of the prevalence of MM.

Furthermore, the estimated proportion of the sample with headaches, eye diseases, musculoskeletal and dermatologic conditions increased more than 40% when the data were combined. Based on a single source, 4 out of these 5 conditions had a higher prevalence according to the Adm database. The results also suggested the potential underestimation of chronic conditions when using one data source. We observed high relative percent differences (60 to 87.7%) for obesity, cancer, hyperlipidemia, gastrointestinal and liver diseases, with 4 out of 5 conditions showing higher SR prevalence. These findings are in line with previous studies reporting inconsistencies in prevalence estimations due to the use of different data sources [23–25, 30, 31, 60]. Based on these results, if one were to use Adm data only, there would be an underestimate of the prevalence of obesity and hyperlipidemia. If one were to choose SR data only, there would be an underestimation of up to 3 times the prevalence of cancer as reported in Adm databases.

Moreover, even when the prevalence estimated with single datasets (not combined sources) was apparently similar (e.g., respiratory tract and eye diseases), the calculated percentage increase obtained when combining both data sources suggests that our data sources identify different cases [48, 58].

As reported in previous studies including older populations, the agreement between data sources for the 17 chronic conditions was highly variable [24, 27, 30, 50]. For some diagnoses, such as diabetes, agreement is good. This may be explained by the health policies aimed at chronic condition management programmes in primary care for diabetes including both health professional and patient participation in treatment management. For other diagnoses, such as cancer and mental health conditions, agreement is poorer. Although associated with a high burden on the health system, the episodic nature of common mental disorders and treatable cancers may have been underreported by patients if interviewed while in remission. Such variability in potential underreporting is an important aspect to be explored in future studies since it may play a central role in patient involvement in health care decision-making. In the particular case of obesity, the low agreement between data sources might have occurred because the cut-off for self-reported obesity (BMI ≥ 30) may not correspond to the one applied in clinical practice where clinicians would be more likely to code a diagnosis of obesity only when it aggravates another condition. Others have suggested that the low agreement may also be a case of less reliable Adm database coding where only the most severe cases of obesity would be recorded [61]. While this latter Canadian study was performed among hospitalized patients, our results support a similar pattern among older adults in the context of primary care. In terms of κ values, our results are very similar to those presented by Fortin and colleagues (2017) [30]. However, according to the literature [22, 49, 50, 60, 62], this coefficient should not be reported or interpreted alone but rather jointly with other indicators. The k statistic takes into account not only the agreement occurring by chance but also the complex influence of the difference in the disagreement cells (BI) and the agreement cells (PI) in a 2 × 2 table [49]. Regardless of the chronic conditions assessed, our results showed that most k coefficients were low (fair to poor) and that BI and PI varied widely and independently of each other. If we contrast pairs of conditions that showed the same k values (e.g., dermatologic conditions and obesity; headaches and arthropathy; cancer and chronic urinary problems), we notice that the other indicators showed different values (Fig. 1; Table 2). Evaluating the set of reported indicators, not only the kappa, (Fig. 1; Table 2), leads to a better understanding of which conditions may be better identified when the different data sources are combined.

The prevalence of self-reported MM was higher than that of the cases identified through Adm data. Combining both databases allowed an increase of 10.6 and 22.4% in the estimation of MM2 and MM3 cases, leading to 95.9% of participants being identified as having two or more chronic conditions. Previous studies focusing on older adults and using patient chart or electronic health records (EHR) in a primary care setting also reported a MM prevalence higher than 90% [17, 63]. Many public health systems in Canada are investing in EHR [64]. Future studies should focus on comparing EHR to datasets derived from combined data sources such as SR and Adm [23, 65]. Rendering this possible requires policies that facilitate data access.

This study also showed an association between a number of sociodemographic and behavioural factors and the presence of MM defined according to different data sources and cut-offs**.** For example, gender was not associated with MM measured with Adm data but was associated with self-reported MM (any cut-off). This finding is in line with the idea presented in previous studies [15, 30, 31] that different population subgroups are captured when different data sources and operational criteria are used to identify MM cases. This may highlight the influence of certain biopsychosocial factors reflected in SR data but less so in Adm data based on physician diagnoses.

### Limitations

The limited number of chronic conditions included in this study (*n* = 17) can be seen as a first limitation. According to the literature, the greater number of co-morbidities, the greater the possibility that MM will be identified [16, 17]. However, there is still no standardized solution as to the ideal size of the list of conditions [10, 15]. Nevertheless, we included 10 of the 11 diagnoses suggested by Diederichs and colleagues [66] as the most frequent diagnoses for people aged 65 years and older and 90% of those recently suggested by Fortin and colleagues [67]. Moreover, the content validity of our chronic condition list, which was influenced by lists proposed by experts in the MM field, may be considered a strength. Second, the use of other self-reported or claim-based information, such as pharmacological or non-pharmacological treatments, laboratory tests and medical procedures, would increase the reliability of diagnosis, but this was not feasible in the current study [5, 43]. The test results received by patients may lead to more accurate self-reports, whereas physicians may add a diagnosis to the visit in view of further testing to confirm or rule out a disorder.

Third, prevalence estimates of certain conditions according to the Adm database might have been overestimated because some codes may indicate a tentative diagnosis. The use of selected algorithms (e.g., at least two outpatient diagnostics within a year), which were not used in this study, might have reduced this potential bias [30, 60, 68]. A previous Canadian study [26], however, showed no benefit in using restrictive criteria for defining a case. Since information about diagnostic codes is not mandatory in the RAMQ medical services claims database, this kind of restriction may lead to an underestimation of cases. Nevertheless, the use of MED-ÉCHO data on hospitalizations may lead to improved sensitivity of measures. Further studies are needed to explore and compare these aspects.

The reasons for the differences in terms of agreement are not straightforward and, based on the information available in this study, can only be hypothesized. The literature proposes several factors influencing agreement. For example, we used a 3 year look-back window, and the use of additional years may have increased the agreement between the two data sources [26, 30, 31, 69]. Furthermore, a relatively low prevalence of certain diseases (e.g., liver diseases and headaches) might affect the kappa coefficients, thus reinforcing the relevance of presenting other agreement measures [60]. Additionally, the results reported based on Adm databases depend on coding accuracy, while those based on SR data depend on respondents’ health literacy and memory [22, 25, 68]. Stigmatization and social desirability can also influence the reporting of certain conditions (e.g., cancer; mental health disorders) [27, 59, 70]. Not using a timeframe as to the presence of a self-reported health condition in our survey question may also have contributed to the low agreement between data sources, especially in the case of episodic chronic conditions such as urinary and dermatologic conditions [60, 69].

The association between sociodemographic and behavioural variables was not consistent for all MM definitions, and only bivariate models were tested. Further research using multivariate models is needed to examine the simultaneous relationship of multiple independent variables and MM according to different definitions. Additionally, future studies using combined data sources should consider an examination of outcomes such as quality of life, functional autonomy and associated health care costs.

Finally, the transferability of results is limited to older adult primary care service users within the context of a universal public health care system. Moreover, more global validation studies concerning Quebec’s health-Adm data are still needed since most of the previous studies were limited to a restricted set of conditions or populations [69, 71–74].

## Conclusion

This is the first study to address, in primary care older adults, the concordance between SR and Adm data in the context of a public health system where residents are covered for all hospitalizations and outpatient consultations with physicians. This study also goes beyond the present literature by simultaneously investigating MM (according to two operational definitions) and chronic condition prevalence. Our findings suggest that no single data source is more valid than another, and we highlight the usefulness of combining two data sources that may, in fact, reflect different patient (SR) and health system (Adm) perspectives. Furthermore, the populations identified by each data source may present different clinical and need profiles. Given the increasing prevalence of MM due to the ageing population, policy and decision makers need to consider both Adm and SR data when estimating population health needs to be able to adequately allocate health and human resources to ensure quality of care and to improve the efficiency of the healthcare system.

## Additional file

Additional file 1: **Table S1.** List of 17 chronic conditions categories and corresponding International Classification of Disease, 9^th^ and 10^th^ Revisions (ICD-9/ICD-10). This table includes all ICD-9 and ICD-10 codes that had been used to extract information from administrative databases. (DOCX 20 kb)

## Data Availability

Requests for access to full anonymized dataset should be addressed to the corresponding author. Participants were not requested to give informed consent for data sharing with other researchers outside the team.

## Abbreviations

Adm: Administrative
BI: Bias index
BMI: Body mass index
CAIQ: Commission d’accès à l’information du Québec
CI: Confidence interval
DSM- 5: Diagnostic and Statistical Manual of Mental Disorders, 5th edition
ED: Emergency department
EHR: Electronic health records
Either: Combined sources
ESA-S: Enquête sur la santé des aînés-Services
ESA-S Q: The ESA-S computer-administered questionnaire
GAD: Generalized anxiety disorder
ICD-10: International Statistical Classification of Diseases and Related Health Problems, 10th revision
ICD-9: International Statistical Classification of Diseases and Related Health Problems, 9th revision
IRCMo: International Research Community on Multimorbidity
MED-ÉCHO: Maintenance et exploitation des données pour l’étude de la clientèle hospitalière
MM: Multimorbidity
MM2: Variable multimorbidity defined as the presence of ≥2 chronic conditions
MM2_Adm: Variable multimorbidity defined as the presence of ≥2 chronic conditions_administrative data only
MM2_Either: Variable multimorbidity defined as the presence of ≥2 chronic conditions_combined data sources
MM2_SR: Variable multimorbidity defined as the presence of ≥2 chronic conditions_ self-reported data only
MM3: Variable multimorbidity defined as the presence of ≥3 chronic conditions
MM3_Adm: Variable multimorbidity defined as the presence of ≥3 chronic conditions_administrative data only
MM3_Either: Variable multimorbidity defined as the presence of ≥3 chronic conditions_combined data sources
MM3_SR: Variable multimorbidity defined as the presence of ≥3 chronic conditions_self-reported data only
MMSE: Mini-Mental State Examination
NA: Negative agreement
NAM: Numéro d’assurance maladie
PA: Positive agreement
PABAK: Prevalence-adjusted and bias-adjusted κ
PI: Prevalence index
RAMQ: Régie de l’Assurance Maladie Québec
SD: Standard deviation
SR: Survey/self-reported
κ: Kappa coefficients
