# Supplementary material for: Measuring multimorbidity in older adults: comparing different data sources
Source: BMC Geriatr. 2019 Jun 14;19:166. doi: 10.1186/s12877-019-1173-4 (PMC6570867; doi:10.1186/s12877-019-1173-4)
Supplement: Supplementary file 1 — Table S1. List of 17 chronic conditions categories and corresponding International Classification of Disease, 9th and 10th Revisions (ICD-9/ICD-10). This table includes all ICD-9 and ICD-10 codes that had been used to extract information from administrative databases. (DOCX 20 kb) [file 12877_2019_1173_MOESM1_ESM.docx]

Manuscript Number: BGTC-D-18-00835

Full Title: MEASURING MULTIMORBIDITY IN OLDER ADULTS: COMPARING DIFFERENT DATA SOURCES

File name: Additional file 1

File format: Table (Additional file 1_ Chronic conditions codes.docx)

Title of data: List of 17 chronic conditions categories and corresponding International Classification of Disease, 9^th^ and 10^th^ Revisions (ICD-9/ICD-10)

Description of data: this table includes all ICD 9 and ICD 10 codes that had been used to extract information from administrative databases.

| **Chronic condition categories** | **DIAGNOSTIC CODES** | |
| --- | --- | --- |
|  | **ICD-9** | **ICD-10** |
| Arthropathy | 714.0; 714.1; 714.2; 714.3; 715.x | M05.0; M05.3; M06.1; M06.9; M15.x-M19.x |
| Cancer | 140.x–149.x, 150.x-159.x; 160.x-165.x; 170.x-176.x; 179.x-189.x; 190.x–199.x; 200.x–209.x; 230.x-234.x | C00.x-C14.x; C15.x-C26.x; C30.x-C39.x; C40.x-C41.x; C43.x-C44.x; C45.x-C49.x; C50.x; C51.x-C58.x; C60.x-C63.x; C64.x-C68.x; C69.x-C75.x; C76.x-C80.x; C81.x-C96.x; C97.x; D00.x-D09.x |
| Cardiovascular disease | 394.0; 394.1; 394.2; 395.1; 395.2; 395.9; 428.x; 412.x; 413.x; 427.3; 440.x - 449.x | I05.0; I05.1; I05.8; I06.1; I06.2; I06.8; I06.9; I50.x; I20.x; I25.2; I48.x; I70.x |
| Chronic urinary problem | 585.x; 593.3; 593.4; 593.5; 593.7; 593.8; 593.9; 595.1; 595.2; 595.9; 597.8; 600.x; 601.1; 601.3; 601.8; 601.9; 602.x | N13.4; N13.5; N13.7; N13.8; N18.1-N18.5; N18.8; N28.8; N28.9; N30.1; N30.2; N30.9; N34.1-N34.3; N40.x; N41.1; N41.3; N41.4; N41.8; N41.9; N42.0; N421; N42.9 |
| Common mental health disorders | 296.x; 300.x; 311.x (excluding 300.3) | F30.x-F48.x, F68.x (excluding F42.x) |
| Dermatologic conditions | 136.0; 454.0; 454.2; 691.8; 692.3; 692.4; 692.5; 692.6; 692.8; 692.9; 694.x; 695.4; 695.8; 696.0; 696.1; 696.2; 697.0; 698.2; 698.3; 701.0; 701.1; 701.3; 701.8; 702.9; 705.8; 706.8; 707.0; 707.1; 707.9; 708.0; 708.8; 709.1; 709.3; 709.8; 757.1; 757.3; 757.9; | L10.x; L12.x; L13.x; L20.x; L23.x; L28.x; L30.1; L40.x; L41.x; L43.x; L50.0; L50.8; L58.1; L85.x; L89.x; L93.x; L94.x; L95.x; L97.x; L98.4; Q80.x; Q81.x; Q82.1; Q82.2; Q82.9; I83.0; I83.2 |
| Diabetes | 250.x | E10.1; E10.2; E10.3; E10.4; E10.5; E10.6; E10.9; E11.0; E11.2; E11.3; E11.4; E11.5; E11.6; E11.9 |
| Eye disease | 365.1; 365.2; 365.3; 365.6; 365.8; 365.9; 366x; 379.3 | H25.x-H28.x; H40.1-H40.6; H40.8; H40.9 |
| Gastrointestinal diseases | 530.8; 531.4; 531.5; 531.6; 531.7; 531.9; 555.x, 556.x, 564.1 | K21.9; K25.4-K25.7; K25.9; K50.1; K50.8; K50.9; K51.0; K51.4; K51.5; K51.8; K51.9; K58.9 |
| Headaches | 307.8; 346.0-346.2; 346.8; 346.9; 784.0 | G43.x; G44.0-G44.3; G44.8 |
| Hyperlipidemia | 272.0-272.4 | E78.0-E78.5 |
| Hypertension | 401.x ; 405.x | I10.x; I15.0 ; I15.8 |
| Liver disease | 571.x | K70.0 ; K70.1 ; K70.3 ; K70.9 ; K73.0 ; K73.2 ; K73.8 ; K73.9 ; K75.4 ; K76.0 ; K76.8 ; K76.9 ; K74.0 ; K74.1 ; K74.3-K74.6 |
| Musculoskeletal conditions | 723.1; 724.1; 724.2; 724.3; 724.4; 724.5; 725.x; 726.0; 726.1; 726.2; 726.3; 726.4; 726.5; 726.6; 726.7; 726.9; 727.0; 727.2; 727.3; 729.0; 729.1; 729.2; 729.4; 729.5 | M25.7; M35.3; M54.1; M54.2; M54.3; M54.5; M54.6; M54.8; M54.9; M60.9; M65.3; M65.4; M65.8; M65.9; M70.0-M70.7; M71.5; M72.9; M75.x; M76.1; M76.2; M76.4; M76.5; M76.6; M76.8 |
| Obesity | 278.0 | E66.0; E66.9 |
| Respiratory tract disease | 491.x; 492.x; 493.x; 496.x | J41.0; J41.1; J41.8; J42.x; J43.9; J44.0; J44.1; J44.9; J45.9 |
| Thyroid problem | 240.x- 246.x | E00.x-E07.x |

REFERENCES:

1. Calderón-Larrañaga A, Vetrano DL, Onder G, Gimeno-Feliu LA, Coscollar-Santaliestra C, Carfí A, et al. Assessing and Measuring Chronic Multimorbidity in the Older Population: A Proposal for Its Operationalization. The Journals of Gerontology Series A: Biological Sciences and Medical Sciences. 2016; :glw233.
2. Fils JM, Penick EC, Nickel EJ, Othmer E, DeSouza C, Gabrielli WF, et al. Minor Versus Major Depression: A Comparative Clinical Study. Prim Care Companion J Clin Psychiatry. 2010. doi:10.4088/PCC.08m00752blu.
3. Fortin M, Almirall J, Nicholson K. Development of a Research Tool to Document Self-Reported Chronic Conditions in Primary Care. Journal of Comorbidity. 2017; 7:117–23.
4. Lesage A, Émond V. Surveillance des troubles mentaux au Québec : prévalence, mortalité et profil d’utilisation des services. Québec, Canada: Institut national de santé publique du Québec.; 2012. <https://www.inspq.qc.ca/pdf/publications/1578_SurvTroublesMentauxQc_PrevalMortaProfilUtiliServices.pdf>.
5. Nicholson K, Terry AL, Fortin M, Williamson T, Bauer M, Thind A. Examining the prevalence and patterns of multimorbidity in Canadian primary healthcare: a methodologic protocol using a national electronic medical record database. Journal of Comorbidity. 2015; 5:150.
6. Public Health Agency of Canada. Life with arthritis in Canada: a personal and public health challenge. Ottawa - Ontario: Public Health Agency of Canada; 2010. <https://www.canada.ca/en/public-health/services/chronic-diseases/arthritis/life-arthritis-canada-a-personal-public-health-challenge.html>.
7. Sadek N, Bona J. Subsyndromal symptomatic depression: A new concept. Depress Anxiety. 2000; 12:30–9.
8. Shields M, Carroll MD, Ogden CL. Adult Obesity Prevalence in Canada and the United States. NCHS Data Brief. 2011; 56:1–8.
